# Metric for Measuring the Effectiveness of Clustering of DNA Microarray Expression

**DOI:** 10.1186/1471-2105-7-S2-S5

**Published:** 2006-09-26

**Authors:** Raja Loganantharaj, Satish Cheepala, John Clifford

**Affiliations:** 1 Bioinformatics Research Lab, University of Louisiana at Lafayette, PO Box 44330, Lafayette, LA 70504, USA; 2 Department of Biochemistry and Molecular Biology, LSU Health Sciences Center and Feist-Weiller Cancer Center, Shreveport, LA 71130, USA

## Abstract

**Background:**

The recent advancement of microarray technology with lower noise and better affordability makes it possible to determine expression of several thousand genes simultaneously. The differentially expressed genes are filtered first and then clustered based on the expression profiles of the genes. A large number of clustering algorithms and distance measuring matrices are proposed in the literature. The popular ones among them include hierarchal clustering and k-means clustering. These algorithms have often used the Euclidian distance or Pearson correlation distance. The biologists or the practitioners are often confused as to which algorithm to use since there is no clear winner among algorithms or among distance measuring metrics. Several validation indices have been proposed in the literature and these are based directly or indirectly on distances; hence a method that uses any of these indices does not relate to any biological features such as biological processes or molecular functions.

**Results:**

In this paper we have proposed a metric to measure the effectiveness of clustering algorithms of genes by computing inter-cluster cohesiveness and as well as the intra-cluster separation with respect to biological features such as biological processes or molecular functions. We have applied this metric to the clusters on the data set that we have created as part of a larger study to determine the cancer suppressive mechanism of a class of chemicals called retinoids.

We have considered hierarchal and k-means clustering with Euclidian and Pearson correlation distances. Our results show that genes of similar expression profiles are more likely to be closely related to biological processes than they are to molecular functions. The findings have been supported by many works in the area of gene clustering.

**Conclusion:**

The best clustering algorithm of genes must achieve cohesiveness within a cluster with respect to some biological features, and as well as maximum separation between clusters in terms of the distribution of genes of a behavioral group across clusters. We claim that our proposed metric is novel in this respect and that it provides a measure of both inter and intra cluster cohesiveness. Best of all, computation of the proposed metric is easy and it provides a single quantitative value, which makes comparison of different algorithms easier. The maximum cluster cohesiveness and the maximum intra-cluster separation are indicated by the metric when its value is 0.

We have demonstrated the metric by applying it to a data set with gene behavioral groupings such as biological process and molecular functions. The metric can be easily extended to other features of a gene such as DNA binding sites and protein-protein interactions of the gene product, special features of the intron-exon structure, promoter characteristics, etc. The metric can also be used in other domains that use two different parametric spaces; one for clustering and the other one for measuring the effectiveness.

## Background

The availability of microarray technology at an affordable price makes it possible to determine expression of several thousand genes simultaneously. For example, the AFFYMETRIX 430 2.0 array contains oligonucleotide probe sets representing approximately 39,000 mouse gene mRNA transcripts. Gene expression levels for a particular tissue or cell type under different conditions are captured by first isolating RNA from the test sample. Through a series of standardized reaction steps, each RNA sample is labeled fluorescently and used to probe an individual chip. Once the expression levels of the genes are quantified under all conditions, the differentially expressed genes are filtered using one of the several methods, such as fold change from one condition to another. Very often the number of differentially expressed genes in a particular comparison are in the order of hundreds.

The differentially expressed genes are then clustered using the expression profiles compared across the different conditions. Clustering is a technique that groups objects of similar features together and it has been studied thoroughly in statistics and data-mining literature [1]. Among many clustering algorithms, hierarchal and k-means clustering algorithms are widely used in microarray analysis [2]. The expression values of genes under *k *different conditions may be viewed as a data point in *k *dimensional space. A clustering algorithm groups nearby data points in *k*-dimensional space together. Several distance measuring metrics have been proposed in the literature and the popular ones among them include Euclidian distance and Pearson correlation distance. Each algorithm clusters the genes differently and the same algorithm may have different results with each different distance metric. With a lack of any guideline for selecting appropriate algorithms and the associated distance metric, biologists and other researchers are confused as to which algorithms and the distance matrices to choose. The problem is further compounded with the influence of data instance over the effectiveness of an algorithm. Visualizing the expression profiles of each cluster for selecting a clustering algorithm is laborious and error prone and can not be done with a large number of genes.

To alleviate the problem in judging the quality of clusters or in validating clusters, several validation methods have been proposed in the literature including c-index [3], Dunn's based index [4], Davies-Bouldin index [5], Silhouette method [6]. Bezdek et al. [7] had compared several indices for their effectiveness in validating clusters and had suggested Dunn's index to be the best among those they have tested. Bolshakova [8] had developed an integrated platform for clustering microarray genes using hierarchal and k-means algorithms and measuring some of these cluster validation indices. All these cluster validation methods directly or indirectly relate the cluster density and separation among different clusters. These measures are generic and are using the same parametric space being used to cluster the objects.

Alternatively, clusters can be validated using its effectiveness in predicting correct membership. Yeung et al. [9] proposed a method called figure of merit for validating clusters based on an estimate of the predictive power of a clustering algorithm. In their approach they apply the clustering algorithm to all the experimental conditions except for one and then use the left out condition to calibrate the predictive power of the clustering algorithm.

None of these methods proposed to validate clusters or to measure the quality of clusters has any bearing on biological interpretations of the clustered genes. Genes that are regulated by the same transcription factors or sets of transcription factors are expected to express similarly under different conditions. Hence, when genes of similar expression patterns are clustered together, it is expected that they share regulation by some of the same transcription factors and that they share function or are involved in some of the same biological processes. In this paper we investigate how to relate the quality of clusters with the expected outcome of clustering: cohesiveness of molecular function or biological processes in each cluster and the separation of biological behaviors among different clusters. We have used GO ontology to abstract the biological processes and molecular functions of genes and use this information to test the proposed metric to measure the effectiveness of clustering in terms of behavioral cohesiveness in clusters. For a lack of a better word, we use *behavior *to refer to either molecular function or biological process in the sequel. Our approach may be viewed as an extension to the recent work of Jakel et al. [10] in which they refer to an external validation. They used cluster selectivity and cluster sensitivity as a measure of external validation.

### Preliminaries

Several algorithms have been used in the literature for clustering DNA microarray expression and we will consider two popular algorithms, namely hierarchal and k-means clustering. We briefly describe each of them.

### Hierarchical clustering

The data points are represented as hierarchical series of nested clusters and this representation has a single cluster at the root level and each branch leads to a cluster from top to leaf node [11]. There are two ways of building hierarchical clustering namely, bottom up and top down. In the bottom up approach every data point is considered to be a cluster and a cluster is merged into another cluster based on their proximity to each other. The proximity measures include single link, average link, complete link and un-weighted pair group. We use *average linkage clustering*, which is defined as the average of all the distances among all the pairs of elements between two clusters, say *m *and *n*. It is represented formally as

Average link distance = Σ(d_ik_|e_i _∈ Cl_m _∧ e_k _∈ Cl_n_)/(|Cl_m_| * |Cl_n_|) where d_ik _is the distance between the elements e_i _of cluster *m *and e_k _of cluster *n*. |Cl_r_| is the size or the total number of genes in cluster Cl_r_.

When a cluster is merged into another cluster, a branch is formed and the process continues until no more individual clusters remain. Once the hierarchical cluster tree is constructed, only one cluster exists at the root level that includes all the genes. As we go down the tree each branch indicates divisions of a cluster into more clusters and the measure of closeness among the clusters are also increasing.

### K-Means Clustering

The data points in m-dimensional space are clustered together into k-groups. The algorithm starts by selecting k-data points randomly and these points are called cluster centers. The distance of each data point, say *i*, to these cluster centers are computed and the data point *i *is associated with the cluster of the closest cluster center. When all the data points are associated with the clusters, the new cluster center is computed and the process of associating data points to the closest cluster center continues until there are no significant changes in the cluster center between iterations.

### Distance measuring metric

The Euclidian distance *d*_*i*,*j *_between a pair of genes, say *g*_*i *_and *g*_*j *_with expression values under *m *conditions is given by

 where *e*_*ir *_the expression value of gene *g*_*i *_under the condition *r*.

The Pearson correlation coefficient between a pair of gene expressions, say *g*_*i *_and *g*_*k*_, is given by

 where σ_i,j _is the covariance of the gene expression of *i *and *j*, and σ_i _and σ_j _are the standard deviation of the expression of gene *i *and gene *j *respectively.

### Gene Ontology

The gene ontology (GO) project [12] provides structured controlled vocabularies to address gene products consistently over several databases including FlyBase (*Drosophila*), the *Saccharomyces *Genome Database (SGD) and the Mouse Genome Database (MGD). The ontology describes gene products in terms of their associated biological processes, cellular components and molecular functions for each annotated gene. Each description of a gene product is arranged in a hierarchy from more general to very specific.

In this work we identify the molecular function and biological process of each gene and use these behaviors to test the proposed metric in assessing the success of a clustering algorithm.

## Results

### Our Approach

The approaches proposed in the literature to access or validate clusters can be broadly classified into measurements that (1) relate to cluster density and cluster separation, or (2) relate to effectiveness of predictability. It is clear that all these matrices are working in the same parametric space and these measurements are very useful if the expectation of a DNA microarray clustering is to serve only to find similarly expressed genes. Unfortunately, biologists typically use clustering as a first step in the process of inferring similar functions or biological processes from each cluster.

We have proposed a metric to measure the effectiveness of clustering DNA microarray expression data with respect to biological processes or molecular functions. To obtain the biological functions of genes we have used gene ontology. Suppose we are interested in measuring the functional cohesiveness of clusters. If a cluster is functionally cohesive, a biologist could infer the function of unclassified genes in the cluster from the known functional annotation of other genes from the same cluster. Therefore, the metric must measure the extent of predictability of genes' function in a cluster. A predictability can conveniently be related to Shannon's information theory [13]. The information content of a cluster reflects its predictability; the higher the value of predictability, the lower the value of information content becomes. We have defined cluster cohesiveness using Shannon's information theory and the details are provided in the section on methods. Clusters are said to be well separated if different clusters are associated with different functions. In other words, if a functional association to a cluster is predictable then the separation of clusters with respect to specific functions becomes better. Here again we can use Shannon's information theory to capture the intra-cluster cohesiveness that reflects cluster separation. The details of the definition and the formulation are given in the section on methods.

Our approach to validate clusters using biological features of genes is an external validation technique and may be viewed as an extension of the recent work of Jakel et al. [10] in which cluster selectivity and cluster sensitivity are used for validation. Unfortunately, the cluster selectivity and sensitivity do not provide an objective means of comparing algorithms for their effectiveness in achieving cohesiveness of biological features. We have proposed cluster cohesiveness and behavioral cohesiveness as a numeric metric to validate clustering algorithms based on a selected biological feature and the details are given in a section on methods.

### Results on applying the metrics

In this paper we have investigated two popular clustering algorithms namely hierarchal and K-means clustering. Both of these methods use distance as a means of clustering genes of similar expression profiles. We have considered the two most often used distance measuring metrics: Euclidean and Pearson correlation distances.

A hierarchal clustering algorithm with Pearson correlation distance produced 4 clusters when applied to the 176 differentially expressed genes when the minimum similarity among clusters was set to 0.825. The dendrograms of the clusters along with a heatmap is shown in Figure 4. This data set was generated in an experiment that is part of a larger study aimed at determining the cancer suppressive mechanism of a class of chemicals called retinoids. When the same algorithm was applied to the differentially expressed genes with Euclidian distance, also with a similarity of 0.825, it produced 8 clusters and the corresponding dendrograms along with a heatmap is shown in Figure 5. Since the hierarchal clustering of the differentially expressed genes has resulted in 4 and 8 clusters, we have applied 4-mean and 8-mean clustering using both distance metrics.

There are altogether 6 outcomes after applying clustering algorithms on the differentially expressed genes: two outcomes by applying hierarchal clustering using each distance metric. 4-means and 8-means clustering algorithms each produces 2 outcomes one for each distance metric. To validate and to rank the outcome, we have applied cluster cohesiveness and behavioral cohesiveness and the results are tabulated in Table 9. The value of cohesiveness ranges from 0, the best, to any other positive number. The smaller the value, the better the cohesiveness becomes and hence the better the clustering. When we compare the cluster cohesiveness of molecular function with that of biological processes, the cohesiveness with respect to biological process consistently outperforms molecular function for each clustering method. This is interpreted to mean that co-expression provides a better indication of co-biological processes than of co-function.

As shown in table 9, 4-means clustering with Euclidian distance provides the best clustering with respect to grouping by biological processes. All the clustering algorithms are performing better when compared to a semi-random distribution of genes in which genes of a behavioral group is uniformly distributed among clusters. The total cohesiveness of functional feature is 32.49 and 54.98 respectively for 4 clusters and 8 clusters when the genes are semi-randomly distributed. This is much higher than the largest total cohesiveness of functional feature of all the tested clustering algorithms with 4 clusters, which is 25.23. Similarly, the highest value of the total cohesiveness of functional feature of the entire tested algorithm is 37.57 which is much smaller than that of a semi-random distribution. The total cohesiveness of biological process is 27.69 and 47.38 respectively for 4 clusters and 8 clusters when the genes are semi-randomly distributed. Similar to the metric for functional grouping, the total cohesiveness of biological process of all the tested algorithms are better than that of a semi-random distribution. The table 9 provides the total cohesiveness of clusters for functional and behavioral features. The gene expression profiles for these clusters are shown in Figure 6, Figure 7, Figure 8 and Figure 9. Out of the four clusters, Figure 6 shows the expression profiles of the genes in cluster 1. The Figures 7, 8 and 9 respectively show the expression profiles of the genes in clusters 2, 3 and 4.

The proposed metric provides a novel approach to gauge the effectiveness of gene clustering by using characteristics such as molecular function and biological processes as a measure of gene closeness. Further, this metric addresses the closeness of function within a cluster and separation of function across clusters. We have illustrated the metric using these functional features. This metric can be easily extended to include other features of genes such as DNA binding sites and protein-protein interactions of the gene products, special features of the intron-exon structure, promoter characteristics, etc. These characteristics make sense for the biologist since they are likely to be closely related to patterns of co-regulation.

Further, this metric addresses the closeness of behavior within a cluster and separation of behavior across clusters. The metric can also be used in another domain that uses two different parametric spaces; one for clustering and the other for measuring the effectiveness.

## Discussion

Most cluster validation methods and techniques proposed in the literature work on a single parametric space; generating and validating the cluster is based on one single parameter such as distance. The proposed metric in this paper works on two different spaces, one for clustering and the other for measuring the effectiveness of the clusters based on biological features. We have considered either molecular functions or biological process for validating the following clustering algorithms: hierarchal clustering and k-means clustering. This work may be considered to be an extension to a recent work on external cluster validation by Jakel et al. [10] in which they have used selectivity and sensitivity of gene function as a measure of validation of clusters. In this paper we have developed a metric using Shannon's information theory to capture cluster cohesiveness and behavioral cohesiveness. Our metric yields a single numeric value that is easy to compute and easy to compare many algorithms for their effectiveness in clustering with respect to a chosen biological feature.

This type of metric is necessary for gene clustering based on expression profiles. Co-regulated genes are often expected to share similar biological processes and similar molecular functions. Further, co-expressed genes are expected to be co-regulated. In gene clustering, genes of similar expression profiles are grouped together in the hope of identifying modes of co-regulation (ie. shared transcription factor binding sites in their promoters).

For a hypothetical discussion, consider four clusters and four functional groups. In an ideal or best situation, genes in each cluster fall exclusively in only one functional group. If we apply our metric to this case, the cluster cohesiveness is 0 and the functional cohesiveness is also 0, as has been predicted by our metric (best clustering occurs when the total cohesiveness is 0). In the worst case, when genes in each cluster are equally distributed among the four functional groups, the total cluster cohesiveness will be 8. Similarly when the genes are equally distributed among the four clusters for each functional group the functional cohesiveness is also 8, resulting in a value of 16 for the total cohesiveness. On the other hand, assume that a specific functional group that is concentrated, say X%, in a specific cluster and the rest of the genes in the cluster are equally divided among the remaining functional groups. As X% increases from 80% to 95% in steps of 5%, a cluster cohesiveness metric reduces in value from 1.039, 0.847, 0.627 to 0.365. When the distribution across clusters in a functional group varies with the same distribution, we will get the same value for the functional cohesiveness metric. As has been illustrated by this numeric example, our metric provides a natural interpretation of the cluster effectiveness and the value it computes.

The results presented in Table 9 are based on the annotated information maintained by the GO ontology database. In the present case, out of 176 genes, the GO ontology assigns 93 molecular functional annotations and 86 biological processes. These results are based on only the annotated genes in our dataset. We assume that the 47% of genes that are not annotated for function and the 51% of genes that are not annotated for biological processes will follow a trend similar to the annotated genes.

## Conclusion

In this paper we addressed the problem faced by practitioners when they cluster the differentially expressed genes based on their profiles using one of several clustering algorithms and one of several distance matrices. We have considered a hierarchal clustering and k-means clustering algorithms with Euclidian distance or Pearson correlation distance in this paper for illustrating the proposed metric. The biologists or the practitioners are often confused as to which algorithm to use since there is no clear winner among algorithms or among distance measuring metrics. Several validation indices have been proposed in the literature and these indices are based directly or indirectly on distances; hence a method that uses any of these indices does

In this paper we have proposed a novel approach to measure the effectiveness of gene clustering. We gained inspiration from Shannon's information theory and have proposed a metric to measure gene cohesiveness and behavioral cohesiveness. Shannon's information theory has been applied to solve a broad class of problems including decision trees, optimization problems, and even generic clustering problems. The cohesiveness is measured in terms of achieving homogeneity of a chosen behavior within a cluster. For genes, the behavior can be either a molecular function or a biological process. A cluster is said to be homogeneous when all the genes of a cluster belongs to only one behavioral group and our metric returns 0, indicating the best cohesiveness. A cluster is said to be behaviorally separated to its maximum when no gene of a particular behavioral group is in other clusters other than the one it is assigned to. In such a case, the behavioral cohesiveness metric returns 0 indicating the best separation. The idealistic situation may not be achieved in gene clustering since one gene may map onto many molecular functions or biological process. Table 2 and Table 4 provide functional and process sharing among the annotated genes.

We have demonstrated the metric by applying it to a data set with gene behavioral groups such as biological process and molecular functions. The metric can be easily extended to other features of a gene such as DNA binding sites and protein-protein interactions of the gene products, and other features of the gene structure. The metric can also be used in other domains that use two different parametric spaces; one for clustering and the other one for measuring the effectiveness.

## Methods

### Data and Pre-processing

We have conducted a DNA microarray experiment using the AFFYMETRIX 430 2.0 array, which contains oligonucleotide probe sets representing approximately 39,000 genes. This experiment is part of a larger study to determine the cancer suppressive mechanism of a class of chemicals called retinoids [14]. The major biologically active retinoid is all-trans retinoic acid (ATRA). We have studied the effects of ATRA on skin cancer prevention using the mouse skin 2-stage chemical carcinogenesis protocol. The mouse skin 2-stage chemical carcinogenesis protocol is one of the best-studied models and most informative with regard to understanding molecular mechanisms of carcinogenesis and identifying chemopreventive agents [15]. Skin tumors can be readily induced in this model by the sequential application of a carcinogen, referred to as the initiation stage, followed by repetitive treatment with a noncarcinogenic tumor promoter, referred to as the promotion stage. The initiation stage, accomplished by a single application of the carcinogen dimethylbenzanthracene (DMBA) to the skin, results in a small subset of keratinocytes (skin cells) carrying a mutation in a critical gene(s). The promotion stage requires repeated (twice weekly) application of tumor promoting agents such as 12-O-tetradecanoylphorbol-13-acetate (TPA) that causes the initiated cells to proliferate, eventually producing tumors. ATRA has been shown to be a highly efficient suppressor of tumor initiation and promotion in this model [16].

Here we describe analysis of the gene expression profiles obtained from microarrays for the following mouse skin samples subjected to the 2-stage protocol for 3 weeks; (1) controls treated with acetone solvent alone, (2) TPA (1 μg/application dissolved in 200 μl acetone), (3) ATRA alone (5 μg/application), and (4) TPA plus ATRA. We chose the 3 week time point in the 2-stage protocol, which is 5–7 weeks prior to the appearance of tumors, in order to identify gene expression changes early in the carcinogenic process that may be influenced by ATRA.

Out of the 39,000 genes on the array, we are interested in those that are upregulated or downregulated by either TPA or ATRA treatment alone compared to controls (≥ 2-fold change or ≤ 0.5 fold change comparing samples 1 to 2 or samples 1 to 3), and which remain unchanged in expression when ATRA and TPA are coadministered compared to controls (fold changes are within 0.834 to 1.2 comparing samples 1 and 4) With this filter, we obtained 192 probe-ids out of which 176 are associated with gene names. These *filtered-genes *were used for further clustering and processing. Expression values of each gene are normalized in order to compensate for the variations of each gene's absolute expression value. Suppose the expression value of a gene, say *g*, under a condition, *i*, is e_gi_. Then the normalized expression value of e_gi _becomes (e_gi _- μ)/σ where μ and σ are respectively the mean and the standard deviation of the expression of the gene *g *over all the conditions.

### Clustering based on GO ontology

**Figure 1:**
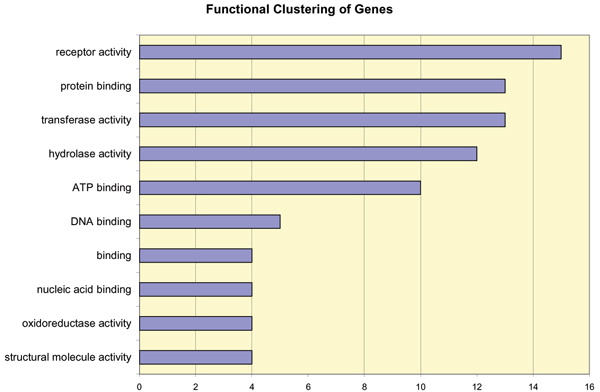
Distribution of molecular function.

Out of the 176 filtered genes, 93 have functional annotations and 86 have biological process annotations from GO ontology. These genes form major functional and biological process clusters. The functional clusters with four or more genes are considered and the details of the clusters are shown in Table 1 and their functional distribution is shown in Figure 1.

A gene may be associated with more than one function and hence may belong to more than one functional group and the number of common genes among these functional groups is shown in Table 2. For example, the entry at the fifth row and third column indicates 2 genes are common in both the functional groups F_6 _and F_3_. The last row shows the total number of genes in a group shared by other functional groups. For example, the last row of third column indicates 2 genes of F_3 _are shared by other functional groups.

**Figure 2:**
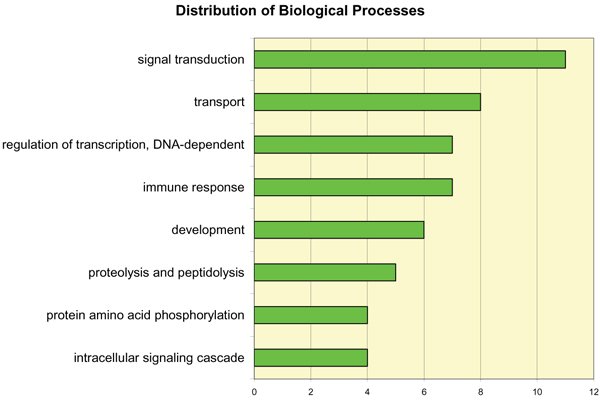
Distribution of Biological Processes.

The clusters of biological processes with eight or more genes are considered and the details of the cluster are shown in Table 3 and the biological processes distribution is shown in Figure 2.

Similar to function, a gene may be associated with more than one biological process and hence may belong to more than one process group and the number of common genes among these groups is shown in Table 4. For example, the entry at the fifth row and forth column indicates that 4 genes are common in both the biological processes groups B_6 _and B_4_.

### Clustering based on gene expression profiles

**Figure 3:**
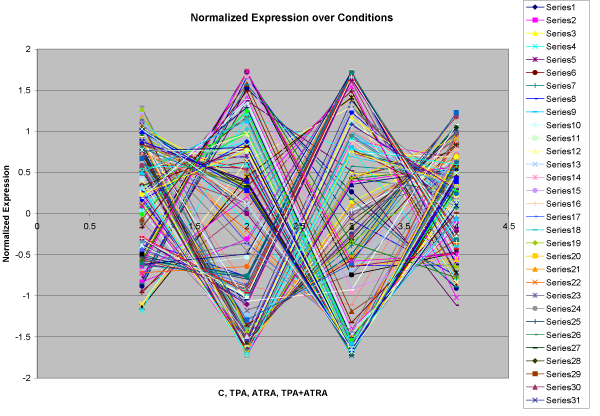
Normalized Expression Profiles of the filtered genes.

**Figure 4:**
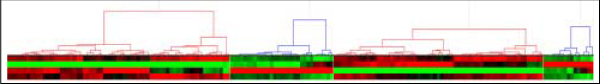
Hierarchal clustering of normalized expression value (Pearson distance) with similarity 0.825.

**Figure 5:**
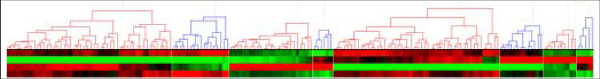
Hierarchal clustering of normalized expression value (Euclidean distance) with similarity 0.825.

**Figure 6:**
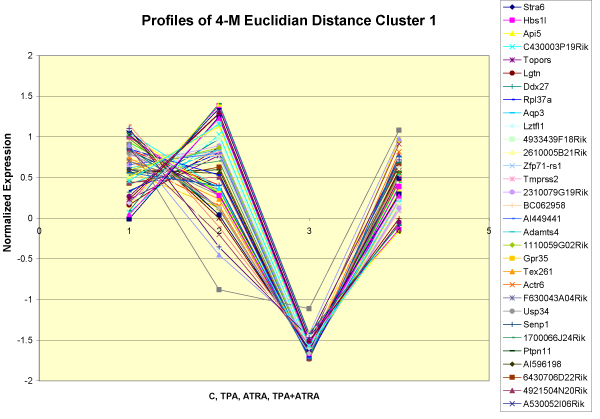
Expression Profiles of genes in cluster 1 of 4-means clustering with Euclidian Distance.

**Figure 7:**
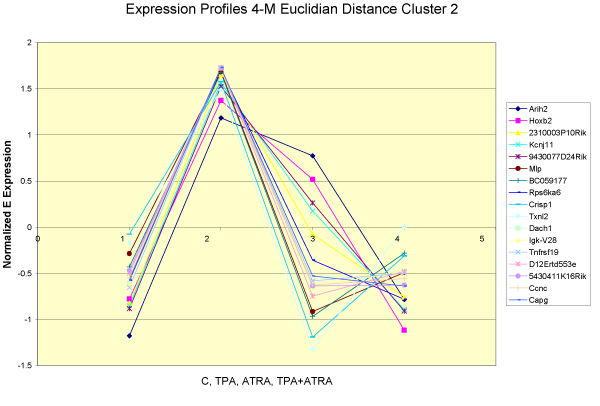
Expression Profiles of genes in cluster 2 of 4-means clustering with Euclidian Distance.

**Figure 8:**
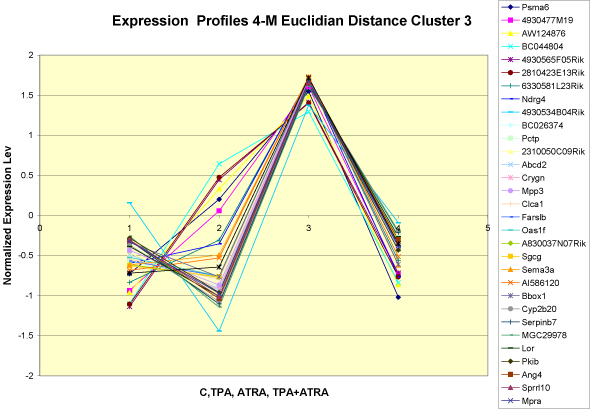
Expression Profiles of genes in cluster 3 of 4-means clustering with Euclidian Distance.

**Figure 9:**
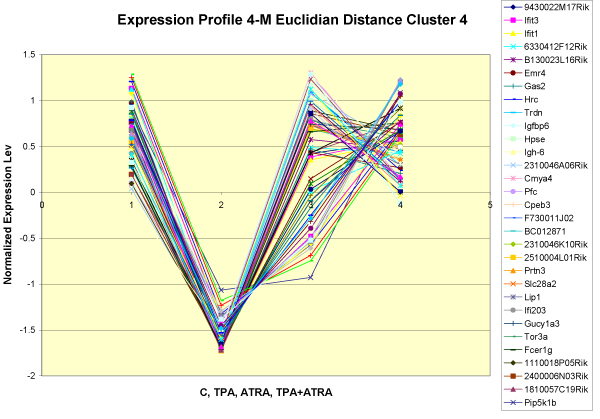
Expression Profiles of genes in cluster 4 of 4-means clustering with Euclidian Distance.

The normalized expression profiles of these 176 filtered genes are shown in Figure 3. We have grouped these genes using hierarchal clustering Explorer Version 3.5 [17] with the average linkage method for each distance metric; namely Euclidian distance and Pearson correlation distance. The outcome of clustering varies with assured minimum similarity and for the experiment we have set the similarity to 0.825.

The hierarchal clustering with Euclidian distance resulted in 8 clusters for the minimum similarity of 0.825 as shown in Figure 2 while the Pearson correlation coefficient with the same similarity resulted in 4 clusters as shown in Figure 3. Since two different distance matrices resulted in 4 and 8 clusters, we have created 4-means and 8-means clusters for each of the distance metrics for comparison purposes.

### Quality of Clustering

The number of clusters and the content in each cluster are dependent on the clustering methods and the metric being used to measure the distance. The quality of a clustering algorithm is proportional to achieving one or both of the following features: (1) maximum density with minimum diversity within a cluster and (2) maximum separation between clusters. The following approaches measure one or both of these features directly or indirectly and have been used to compare different clustering methods: Dunn's, Davies-Bouldin, Silhouette, C, Goodman-Kruskal, Isolation, Jaccard and Rand. Bolshakova et al. [18] have used some of these indices to compare different proximity measures of hierarchal clustering. While these approaches are excellent to get an assessment of inter-cluster cohesiveness and intra-cluster separation, these methods will not be useful for measuring the cluster quality of genes since distance between expression profiles does not map onto gene behaviors such as molecular function or molecular processes.

Recently, Speer et al. [19] have used GO functional annotations to cluster genes using minimum spanning tree with single link proximity measures. Incompatible links in the minimum spanning tree are removed to form a minimum number of spanning trees and each spanning tree forms a cluster. They have applied the Davies-Bouldin index for estimating the quality of clusters.

All the approaches are based directly or indirectly on the information used for clustering and these methods ignore the intended purpose of gene clustering. DNA microarray expression data are clustered based on their expression profiles, often with the expectation that genes with similar behavioral features group together. In an ideal situation, one to one mapping from a cluster to a behavioral group is expected. We propose a method to measure the degree of achieving cohesiveness of behavior among the genes within a cluster.

Suppose, *n *annotated genes are clustered into *m *groups based on their gene expression profiles. Assume that these *n *genes form *k *behavioral clusters based on the GO annotation. **Our idea for a metric to measure gene clustering is based on behavioral homogeneity within a cluster and maximum separation of behavior across clusters.**

Let *p*_*ir *_be the probability of selecting a gene of behavioral group *i *within a cluster *r*. Let *n*_*i *_be the number of genes of behavior group *i *in cluster *r *that has total of *n*_*r *_genes. Then *p*_*ir *_= *n*_*i*_/*n*_*r *_and Σ*p*_*ir *_= 1 over all the behavioral groups. We model the behavioral cohesiveness within a cluster using Shannon's information theory. Higher value of cohesiveness is measured by a high degree of certainty that the genes in a cluster belongs to a behavioral group. We define the cohesiveness of a cluster as the information content of a cluster and it is defined by the following formula.





We will define a measure that maximizes the separation of behavior across different clusters. Let *b*_*ir *_be the probability of selecting a gene of behavioral group *i *in cluster *r *among all the genes belonging to the behavioral group *i*. Suppose, *n*_*ir *_is the number of genes of behavior group *i *in cluster *r *and the total number of genes in the behavioral group *i *is *N*_*i*_. Then *b*_*ir *_= *n*_*ir*_/*N*_*i *_and Σ*b*_*ir *_= 1 over all the clusters. The information content of a behavioral group *i *in all the clusters reflects the cohesiveness of the behavioral group and thereby indicates the separation of behavior between clusters.





The quality of clustering is measured by combining the total cluster cohesiveness and behavioral group cohesiveness as has been defined above. The lower the total value of cohesiveness of clusters and behavioral groups, the better the quality of clusters becomes.

The metric that we have proposed provides a quantitative measure to rank clustering algorithms based on biological validity measures such as molecular function or biological processes. Further the metric is easy to compute and easy to understand conceptually. For comparison, let us consider a worst case scenario in which each behavioral group is equally distributed among all the clusters, say *k*. Suppose, we have *n *behavioral groups and the behavioral group *i *has |g_i_| genes. *P*_*ir*_, the probability of selecting a gene of behavioral group *i *within a cluster *r*, is given by



Note that the value of *p*_*ir *_depends only on the number of genes in each behavioral group and it is independent of a particular cluster. Using the formula 2, we can compute the total cohesiveness of all the clusters.

*B*_*ir*_, the probability of selecting a gene of behavioral group *i *in cluster *r *among *k *clusters, is *1/k *since we are assuming that genes of each behavioral groups are equally distributed among the *k *clusters. When we apply the value of *b*_*ir *_on formula 4, the total behavioral group cohesiveness becomes – n * log(1/k). Thus, we compute the inter cluster cohesiveness and behavioral cohesiveness for the given experimental data set when each behavioral group is equally distributed among the clusters.

### Application

Clustering of the normalized expression profiles of the 176 filtered genes using hierarchal clustering Explorer Version 3.5 [17] with the average linkage method resulted in eight and four clusters respectively, when using Euclidian and Pearson correlation distance. The outcome of clustering varies with assured minimum similarity. For this experiment we have set the similarity to 0.825. The hierarchal clustering with Euclidian distance resulted in 8 clusters for the minimum similarity of 0.825 as shown in Figure 2 while the algorithm with Pearson correlation coefficient distance with the same similarity resulted in 4 clusters as shown in Figure 3. Since Pearson correlation distance and Euclidian distance, respectively, resulted in 4 and 8 clusters, we have created 4-means and 8-means clusters for each of the distance metrics for comparison. The genes in each cluster were further clustered based on their behavioral groups such as biological processes and molecular functions from the GO ontology. The genes were distributed among ten functional groups and among eight biological processes. The details of the distributions are shown in Table 5, Table 6, Table 7 and Table 8.

The metric that we propose in this paper helps to obtain the best gene clustering algorithm that maximizes the cluster cohesiveness and behavioral cohesiveness across clusters. We have computed the cohesiveness for each clustering algorithm and for each distance measuring metric and the results are shown in Table 9. The 4-means clustering with Euclidean distance, as well as hierarchal clustering with Pearson correlation distance, seem to provide better clusters for grouping genes with similar biological processes. On the other hand, 4-means clustering with Pearson correlation coefficient distance seems to provide the best clustering for grouping genes with similar functions.

## Authors' contributions

RL has developed and implemented algorithms, proposed the metric and performed the analysis. SC has conducted the animal experiments and collected the date that was used in the paper. JC has designed and directed the animal experiments and provided the biological interpretation of the results. RL and JC have equally worked on organizing and presenting the materials.

## Figures and Tables

**Table 1:** Molecular functional group among the 93 annotated genes

**Molecular Function**	**Number of Genes**	**Functional Group**
structural molecule activity	4	F_1_
oxidoreductase activity	4	F_2_
nucleic acid binding	4	F_3_
binding	4	F_4_
DNA binding	5	F_5_
ATP binding	10	F_6_
hydrolase activity	12	F_7_
transferase activity	13	F_8_
protein binding	13	F_9_
receptor activity	15	F_10_

**Table 2:** Common genes among the functional groups

	**F1**	**F2**	**F3**	**F4**	**F5**	**F6**	**F7**	**F8**	**F9**
F2	0								
F3	0								
F4	0	1							
F5	0								
F6	0		2	1					
F7	0		1			2			
F8	0		1			6			
F9	2	1		2	1	1	1	1	
F10	1					2	1	2	2
Total-shared	2	1	2	2	1	9	1	3	2

**Table 3:** biological processes group among the 93 genes

**Biological Processes**	**Number of Genes**	**Processes Group**
intracellular signaling cascade	4	B1
protein amino acid phosphorylation	4	B2
proteolysis and peptidolysis	5	B3
development	6	B4
immune response	7	B5
regulation of transcription, DNA-dependent	7	B6
transport	8	B7
signal transduction	11	B8

**Table 4:** Common genes among the biological process groups

	**B1**	**B2**	**B3**	**B4**	**B5**	**B6**	**B7**
B2							
B3							
B4							
B5							
B6				4			
B7							
B8	2						

**Table 5:** Distribution of genes among different major functional groups in each clusters generated by different clustering algorithms.

		**F_1_**	**F_2_**	**F_3_**	**F_4_**	**F_5_**	**F_6_**	**F_7_**	**F_8_**	**F_9_**	**F_10_**
4-means clustering	Euclidian	1	1	2	0	2	3	6	4	2	6
		0	0	0	0	2	1	0	1	1	1
		1	1	1	0	0	2	4	2	2	1
		2	2	1	4	1	4	2	6	8	5
	
	Pearson correlation	2	2	1	4	1	3	2	5	8	5
		1	1	2	0	2	3	6	4	2	6
		0	0	0	0	2	1	0	1	1	1
		1	1	1	0	0	3	4	3	2	1

Hierarchal Clustering	Euclidian	2	0	1	3	1	4	2	3	5	5
		0	2	0	1	0	0	0	3	3	0
		1	1	1	0	0	2	3	2	2	1
		0	0	0	0	0	0	1	0	0	0
		1	1	2	0	1	1	6	1	2	5
		0	0	0	0	1	2	0	3	0	1
		0	0	0	0	1	1	0	1	1	1
		0	0	0	0	1	0	0	0	0	0
	
	Pearson correlation	2	2	1	4	1	4	2	6	8	5
		1	1	1	0	0	2	4	2	2	1
		1	1	2	0	2	3	6	4	2	6
		0	0	0	0	2	1	0	1	1	1

**Table 6:** Distribution of genes among different major functional groups in each clusters generated by 8-means clustering algorithms.

		**F_1_**	**F_2_**	**F_3_**	**F_4_**	**F_5_**	**F_6_**	**F_7_**	**F**_8_	**F**_9_	**F**_10_
8-means clustering	Euclidian	0	0	0	3	1	1	0	0	1	1
		0	2	0	1	0	0	0	3	3	0
		1	1	1	0	1	0	3	1	2	5
		0	0	0	0	1	0	1	0	0	0
		0	0	1	0	1	3	3	3	0	1
		0	0	0	0	1	1	0	1	1	1
		2	0	1	0	0	3	2	3	4	4
		1	1	1	0	0	2	3	2	2	1
	
	Pearson correlation	1	1	0	0	0	0	2	1	1	4
		0	0	2	0	1	1	4	0	1	1
		0	0	0	0	2	1	0	1	1	1
		1	1	1	0	0	2	3	2	2	1
		2	0	1	2	1	3	2	3	4	5
		0	0	0	0	1	2	0	3	0	1
		0	0	0	0	0	0	1	0	0	0
		0	2	0	2	0	1	0	3	4	0

**Table 7:** Distribution of genes among different major biological processes groups in each clusters generated by different clustering algorithms.

		**B_1_**	**B_2_**	**B_3_**	**B_4_**	**B_5_**	**B_6_**	**B_7_**	**B_8_**
4-means clustering	Euclidian	2	2	3	1	0	2	3	4
		0	1	0	2	0	3	1	0
		1	0	0	1	0	1	3	1
		1	1	2	2	7	1	1	4
	
	Pearson correlation	1	1	2	2	6	1	1	4
		2	2	3	1	0	2	3	4
		0	1	0	2	0	3	1	0
		1	0	0	1	1	1	3	1

Hierarchal Clustering	Euclidian	1	1	1	2	6	1	1	4
		0	0	1	0	1	0	0	0
		1	0	0	1	0	1	3	1
		0	0	0	0	0	0	0	0
		2	0	3	0	0	1	3	4
		0	2	0	1	0	1	0	0
		0	1	0	1	0	2	0	0
		0	0	0	1	0	1	1	0
	
	Pearson correlation	1	1	2	2	7	1	1	4
		1	0	0	1	0	1	3	1
		2	2	3	1	0	2	3	4
		0	1	0	2	0	3	1	0

**Table 8:** Distribution of genes among different major functional groups in each clusters generated by 8-means clustering algorithms.

		**B_1_**	**B_2_**	**B_3_**	**B_4_**	**B_5_**	**B_6_**	**B_7_**	**B_8_**
8-means clustering	Euclidian	0	0	0	1	2	1	0	2
		0	0	1	0	1	0	0	0
		2	0	1	0	0	1	2	4
		0	0	0	1	0	1	0	0
		0	2	2	1	0	1	1	0
		0	1	0	1	0	2	1	0
		1	1	1	1	4	0	1	2
		1	0	0	1	0	1	3	1
	
	Pearson correlation	1	0	1	0	0	0	2	3
		1	0	2	0	0	1	1	1
		0	1	0	2	0	3	1	0
		1	0	0	1	0	1	3	1
		1	1	1	2	6	1	1	3
		0	2	0	1	0	1	0	0
		0	0	0	0	0	0	0	0
		0	0	1	0	1	0	0	1

**Table 9:** The result of applying the metrics to clustering algorithms

		**Cluster Cohesiveness**		**Behavioral grp. Cohesiveness**		**Total**	
		Funct.	Proc.	Funct.	Proc.	Funct.	Proc.

4-means	Euclid..	11.01	9.26	14.23	10.94	25.23	20.19
	Pearson	10.99	9.59	14.36	11.53	24.35	21.12
8-means	Euclid..	17.99	14.88	19.59	15.40	37.57	30.28
	Pearson	16.80	13.76	19.01	14.33	35.81	28.09
Hierarchal clustering	Euclid..	14.67	14.5	17.98	12.94	32.66	25.44
	Pearson	11.01	9.26	14.23	10.93	25.23	20.19

## References

[B1] Speed TP (2003). Statistical analysis of gene expression microarray data.

[B2] Nuber UA (2005). DNA microarrays.

[B3] Hubert L, Schultz J (1976). Quadratic assignment as a general data-analysis strategy. British Journal of Mathematical and Statistical Psychologie.

[B4] Dunn JC (1974). Well separated clusters and optimal fuzzy partitions. Journal of Cybernetics.

[B5] Davies DL, Bouldin DW (1979). A cluster separation measure. IEEE Trans Pattern Anal Machine Intelligence.

[B6] Rousseeuw PJ (1987). Silhouettes: a graphical aid to the interpretation and validation of cluster analysis. Journal of Computational and Applied Mathematics.

[B7] Bezdek JC, Pal NR (1998). Some New Indexes of Cluster Validity. IEEE TRANSACTIONS ON Systems, Man and Cybernetics.

[B8] Bolshakova N, Azuaje F (2003). Machaon CVE: cluster validation for gene expression data. Bioinformatics.

[B9] Yeung KY, Haynor DR, Ruzzo WL (2001). Validating clustering for gene expression data. Bioinformatics.

[B10] Jäkel J, Nöllenburg M (2004). Validation in the Cluster Analysis of Gene Expression Data. Workshop on Fuzzy-Systeme and Computational Intelligence: November 10–12 2004.

[B11] Eisen MB Gene Cluster. Hierarchical clustering, self-organizing maps (SOMs), k-means clustering, principal component analysis.

[B12] go-ontology the gene ontology. http://www.geneontology.org/.

[B13] Shannon CE (1948). A mathematical theory of communication. Bell System Technical Journal.

[B14] Xu CS H, McCauley E, Coombes K, Xiao L, Fischer SM, Clifford JL (2006). Chemoprevention of skin carcinogenesis by phenylretinamides: retinoid receptor independent tumor suppression. Clinical Cancer Research.

[B15] DiGiovanni J (1992). Multistage carcinogenesis in mouse skin. Pharmacol Ther.

[B16] Verma AK (1987). Inhibition of both stage I and stage II mouse skin tumour promotion by retinoic acid and the dependence of inhibition of tumor promotion on the duration of retinoic acid treatment. Cancer Research.

[B17] Seo J, Gordish-Dressman H, Hoffman EP (2006). An interactive power analysis tool for microarray hypothesis testing and generation. Bioinformatics.

[B18] Bolshakova N, Azuaje F, Cunningham P (2004). An integrated tool for microarray data clustering and cluster validity assessment. Proceedings of the 2004 ACM Symposium on Applied Computing (SAC): March 14–17 2004: ACM.

[B19] Speer N, Spieth C, Zell A (2004). A Memetic Clustering Algorithm for the Functional Partition of Genes Based on the Gene Ontology. Proceedings of the 2004 IEEE Symposium on Computational Intelligence in Bioinformatics and Computational Biology (CIBCB 2004).

